# Cold Applications in Pneumonia and Pleurisy

**Published:** 1857-11

**Authors:** 


*Cold Applications in Pneumonia and Pleurisy.*—Prof. Niemeyer, in his contributions to clinical medicine, expresses his opinion, based upon numerous observations, that in Pneumonia and Pleurisy, cold applications (around the chest and back) are not only unaccompanied by danger, but as efficient here as in inflammation of external organs. They produce great relief, and the patients, even children, ask for their renewal when they have become warm. Dr. N. saw, under this treatment, the exudation cease earlier, the fever extinguished sooner, and the patients returned to their work on the seventh or eighth day, while he never observed metastatis, or the consequence of cold from this treatment. In the latter stages of these diseases Dr. N. gives preparations of iron with great success, on the theory adopted by distinguished pathologists that the blood-corpuscles are materially diminished in these diseases. (I have seen very good effects from the tinct. ferri muriat. in capillary bronchitis and pneumonia accompanying measles.—TRANS.) (*Medic. Nenigk.*)—*Med. & Surg. Reporter.*

